# Bioaccumulation of Arsenic, Cadmium, Chromium, Cobalt, Copper, and Zinc in *Uroteuthis edulis* from the East China Sea

**DOI:** 10.3390/toxics12070496

**Published:** 2024-07-06

**Authors:** Mengqi Li, Baihao Zhang, Zhou Fang

**Affiliations:** 1 College of Marine Living Resource Sciences and Management, Shanghai Ocean University, Shanghai 201306, China; m220200735@st.shou.edu.cn (M.L.); baihao_zhang@163.com (B.Z.); 2 National Engineering Research Center for Oceanic Fisheries, Shanghai Ocean University, Shanghai 201306, China; 3 Key Laboratory of Sustainable Exploitation of Oceanic Fisheries Resources, Ministry of Education, Shanghai Ocean University, Shanghai 201306, China; 4 Key Laboratory of Oceanic Fisheries Exploration, Ministry of Agriculture and Rural Affairs, Shanghai 201306, China; 5 Scientific Observation and Experimental Station of Oceanic Fishery Resources, Ministry of Agriculture and Rural Affairs, Shanghai 201306, China

**Keywords:** squids, trace elements, distribution in tissues

## Abstract

In this study, the concentrations of trace elements (TEs) in *Uroteuthis edulis* caught from the East China Sea were determined. There were significant differences between TE concentrations in different body parts. Cu, Zn, and Cd were the most concentrated in the digestive glands and the concentrations of Cr and Co were highest in the gills. No significant differences in concentrations were shown between these tissues. In the four tissues analyzed, the mantle recorded the highest proportion of elemental load, while the digestive glands and gills had the lowest proportions. After maturity, TEs in the mantle showed no significant differences. In the digestive gland, the concentrations of all elements, except Zn, were significantly increased. The gonads illustrated apparent increases in the concentrations of Cr, Cu, and As. In the gills, the concentrations of Co and As were markedly increased.

## 1. Introduction

Trace elements (TEs) are defined as metallic elements with a density greater than 5.0 g/cm^3^ [1]. From the perspective of environmental pollution, TEs include certain non-metallic elements such as Arsenic (As) and Selenium (Se) [1]. Approximately 45 types of TEs exist in nature. According to the requirements for physiological function, they can be classified into two categories. The first category includes essential elements and micronutrients such as Copper (Cu) and Zinc (Zn), which are usually necessary for physiological functions [2,3]. The deficiency of these elements affects individual growth, but an excessive intake also has adverse effects. The second category includes nonessential elements, such as Cadmium (Cd) and Lead (Pb), which are nonessential for physiological function and have obvious toxicity. They may be transferred to the food chain in various ways and via different media.

Seawater and marine sediments are significant sources of the pollutants that accumulate in marine organisms. Toxic TE pollutants are absorbed from seawater and marine sediments through terrestrial and aerial pathways. TEs accumulate in marine organisms and are then combined with proteins and enzymes to form toxic substances. In addition, TEs can be transferred to organisms at higher nutritional levels and to human beings via the food chain [4,5], thus posing serious threats to marine ecosystems and humans. Above-standard TE pollutants have led to serious public health incidents such as the “Itai-Itai disease” event caused by Cd poisoning in 1931 and the “Minamata disease” event caused by Hg poisoning in 1956 [6,7,8,9]. According to previous studies, Hg, Cd, Pb, and the semi-metallic element As are the main TEs that have serious impacts on marine life and humans. These elements are certified as important indicators for the detection of “limited toxic and harmful substances” in drinking water products [6,10]. Therefore, it is important to investigate the accumulation of TEs in important economic species in the ocean.

The East China Sea (ECS) is located in the south of the estuary of the Yangtze River and the east of Eurasia. As a marginal sea on the western side of the Pacific Ocean, it covers approximately 700,000 square kilometers [11,12]. Its complex ocean current circulation system supports the reproduction of various fish species; hence, it is one of the most important fishing grounds in China [13]. The inshore fishing grounds of ECS are adjacent to developed regions like Shanghai, Jiangsu, Zhejiang, and Fujian. Consequently, the sediments in the region are influenced by both the Yangtze River and human activities [14,15,16], resulting in high contents of trace elements and severe pollution [15]. However, as one of the most important fishing grounds in China, the ECS is a habitat for many marine organisms and is abundant in nutrients. The pollutants entering the ocean affect the growth and development of marine organisms to a certain degree and ultimately change human health via the food chain.

The squid, *Uroteuthis edulis* (*U. edulis*), is a coastal warm-temperate shallow-water species widely distributed in the northwestern Pacific Ocean and along the eastern coast of Africa [17,18]. In the East China Sea, *U. edulis* is one of the most important economic species; thus, it is important to explore the accumulation of TEs in *U. edulis*. The concentrations of trace elements in fish and shellfish in most seas have been investigated in recent years; however, a similar study on cephalopods in the ECS has seldom been reported [19,20]. A cephalopod is a type of invertebrate with a fast growth rate and a short lifespan and is extremely sensitive to trace element pollutants [21,22]. *U. edulis* prey mainly on crustaceans and small fishes and easily accumulate TEs. *U. edulis* is in the intermediate nutritional level [23], so it is a significant species in food chains. Studies on trace element accumulation have increased in recent years and have mostly focused on food science or environmental aspects. In food science research [24], the concentrations of TEs in the muscular tissues of the target organism were used to assess their edibility. In environmental science research, different cephalopods, including those from Loliginidae across different marine areas, were explored to analyze the effect of location on TE concentrations in the tissues of target organisms [24,25]. *U. edulis* is one of the most important economic species, with an annual catch of 5000~15,000 tons; therefore, understanding the distribution of microelements in *U. edulis* can facilitate effective response to risks and help maintain social stability [21,22]. The concentrations of TEs in the muscular tissues of cephalopods have been measured in most studies; however, other tissues such as gonads and internal organs with significant accumulation of TEs and potential correlation with the enrichment pattern of muscles have not been explored [26,27]. Additionally, the accumulation, transfer, and factors influencing TEs in different tissues of squids from Loliginidae are seldom reported.

This study aims to analyze the distribution of trace elements in different tissues of *U. edulis* and explore the correlation between trace elements in various tissues. This study will provide crucial data and a scientific basis for a better understanding of the enrichment characteristics of TEs in cephalopod species.

## 2. Materials and Methods

### 2.1. Sample Collection

**Figure 1:**
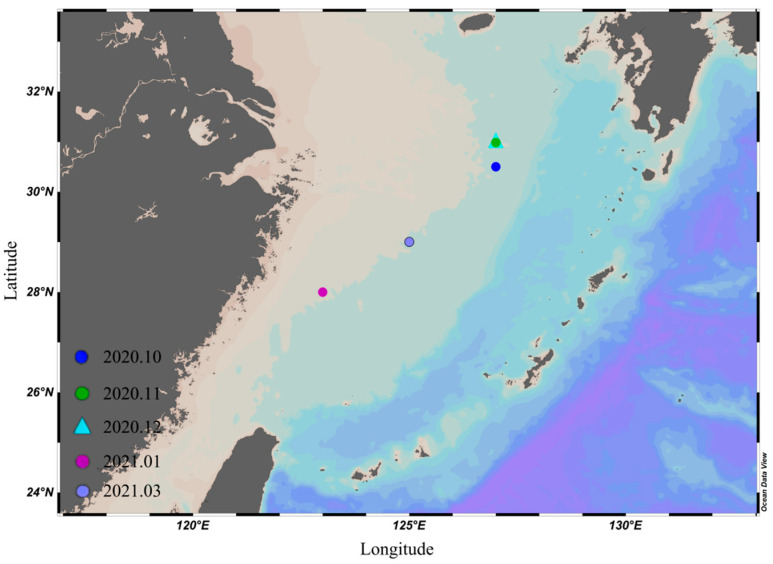
Sampling locations of *Uroteuthis edulis* in the East China Sea. The blue point represents the sampling locations in October 2020; the green point represents the sampling locations in November 2020; the cyan triangle represents the sampling locations in December 2020; the pink point represents the sampling locations in January 2021; the purple point represents the sampling locations in March 2021.

Sampling areas were located in the East China Sea (123° E–127.5° E, 28° N–31.5° N). The samples were caught by the trawler “Zhejiang Lingyu 23860” (Figure 1) and about 50 samples were obtained each month. In this study, 289 samples of *U. edulis* were obtained. All the samples were immediately frozen, transported to the laboratory, and stored at −20 °C.

### 2.2. Sample Processing

The samples of *U. edulis* were thawed at room temperature in the laboratory. All samples were numbered, arranged in the trays, and photographed, and the mantle length and body weight of samples were measured [28]. After the biological data were recorded, the samples were dissected to obtain various tissues including mantle, digestive gland, gonad, and gill. In each month, 8 samples were randomly selected, including 4 mature individuals and 4 immature individuals. After the same treatment, the differences in the contents of trace elements in the tissues of *U. edulis* before and after gonad maturation were analyzed. Finally, the samples were stored in a −20 °C freezer for the subsequent analysis of trace elements [28].

### 2.3. Materials and Methods

Prior to the formal experiment, the scallop standard (GBW10024) was used to finish the pre-experiment, which was utilized to determine the trace elements. The ratio of HNO3 to H2O2, the heating program, and the duration were adjusted, and the experiment was repeated six times to compare recovery rates. The scheme with the highest recovery rate was ultimately selected for the experimental plan. The recovery rates of the six trace elements were all within the norms of chemical testing methods (Table 1). Unfortunately, due to the limited number of samples, the samples were not available in duplicates or triplicates.

#### 2.3.1. Sample Collection

To reduce the interferences from the external environment and residues in the digestion tube, all containers such as polytetrafluoroethylene beakers and digestion tubes had to be washed with acids and rinsed with ultra-pure water. Firstly, containers were soaked in 20% nitric acid (GR grade) for 72 h; then, they were washed repeatedly with clean water; finally, they were rinsed with ultra-pure water. Afterward, containers were dried at 180 °C in a drying oven for 3 h and then cooled to room temperature for use.

#### 2.3.2. Tissue Pre-Treatment

After the samples of *U. edulis* were thawed at room temperature in the laboratory, an appropriate amount of four different tissue samples were weighed and placed in a cryogenic vial for 72 h freeze-drying at −80 °C. After freeze-drying, the samples were ground with agate mortar and then a portion of samples (0.5 ± 0.0005 g) were weighed in an analytical balance with an accuracy of 0.0001 g.

Before the measurements of the trace elements were conducted, the samples were digested to destroy the organic matters and remove the interfering compounds according to the Chinese National Standard GB5009.268-2016 “Food Safety National Standard Determination of Multi-elements in Food” (GB 5009.268-2016). The HNO_3_(65%)-H_2_O_2_(30%) digestion and microwave solver digestion methods were used.

A high concentration of HNO_3_ might damage measurement instruments, so it was necessary to carry out acid-reducing operations. After digestion, all the organic matters were decomposed, but some insoluble substances, such as silicon dioxide, remained, particularly those in the gills. These impurities had to be removed from the solution so as to prevent the blockage of the nebulizer during detection. Subsequently, the samples were filtered through a 25 mm polyvinylidene fluoride (PVDF) 0.22 μm filter membrane, transferred into 2 mL centrifuge tubes, and stored at room temperature for elemental analysis. Detailed information, sample usage, and operation steps are shown in GB 5009.268-2016.

#### 2.3.3. Instrumental Detection of Trace Elements in Tissue Samples

After the pre-treatments, the samples were analyzed with inductivity-coupled plasma–mass spectrometry (ICP-MS) at the trace element detection laboratory of Shanghai Ocean University. The elements analyzed include Chromium (Cr), Cobalt (Co), Copper (Cu), Zinc (Zn), Arsensic (As), and Cadmium (Cd), leading to a total of six elements in this study. Before testing, a proper concentration of the mixed standard solution for multi-element ICP-MS was required to formulate the standard curve. In this study, the concentrations of the standard substance were selected to be 0.01, 0.05, 0.1, 0.5, 1, 2, 5, 10, 50, 100, and 500 ppm. HNO_3_ solution (1%) was used as a blank sample.

Based on the comparison of the signal intensity of elements from both the test solution and the standard solution, the contents of the trace elements in the specimens (ppm) were calculated as follows:

(1) $$\mathrm{Heavy}\;\mathrm{mental}\;\mathrm{concentration}=\frac{\left(C-C_{0}\right)\times V}{W\times 1000}$$

where *C* indicates the concentration of a trace elements in the measured liquid obtained from the standard curve (μg/mL); *Co* indicates trace elements concentration in the blank detection liquid obtained from the standard curve (μg/mL); *V* indicates the final volume of the substance (mL) (in this study, it was 25 mL); *W* indicates the weight of the *U. edulis* sample (g, dry weight) (in this study, it was 0.5 g).

Finally, the outliers in the results were removed and the data analysis was performed.

#### 2.3.4. Data Analysis

The concentrations of Cr, Co, Cu, Zn, As, and Cd in the mantle, digestive glands, gonads, and gills were displayed in box–scatter composite plots. The Shapiro–Wilk test method and variance homogeneity test (F-test) were used to test the data. If both tests were satisfied, then a one-way ANOVA (one-way analysis of variance) was used to analyze the differences among elements in each tissue and the differences of a single element in different tissues. If the data did not follow a normal distribution or meet the condition of variance homogeneity, the Kruskal–Wallis H method was used to test the data. For the correlational analysis among different elements in each tissue and the individual elements in the different tissues, the Spearman rank correlation coefficient was employed. In order to analyze trace elements concentrations before and after gonad maturation, two sample groups were set. Therefore, if the data did not follow a normal distribution or meet the condition of variance homogeneity, then the Mann–Whitney U-test method was chosen to test the differences in the concentration of each element in various tissues before and after maturity. Data analysis and plotting were performed with R language, Excel 2020, and Origin 2021.

## 3. Results

### 3.1. TEs Concentrations

**Figure 2:**
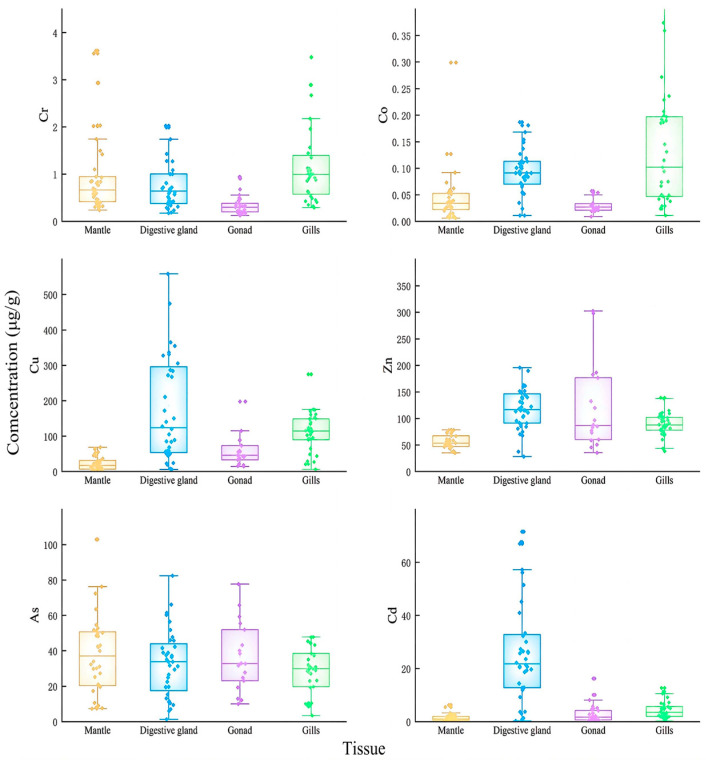
Contents (μg/g (μg/g dry weight)) of selected metals in the tissues of *Uroteuthis edulis*. Yellow represents the mantle; blue represents the digestive gland; purple represents the gonads; green represents gills.

In this study, the mantle length, body weight, and sexual maturity showed significant differences among the samples caught in the same month. Therefore, the box-and-dot composite plot was used to fully depict the distribution differences in trace elements concentrations in the tissue samples of *U. edulis*. The results of Cr, Co, Cu, Zn, As, and Cd in the mantle, digestive gland, gonads, and gills of *U. edulis* are shown in Figure 2.

The concentrations of Cu and Zn were significantly higher than those of other elements (Figure 2). The concentration of Cu was the highest and exceeded 600 μg/g in the digestive gland of one sample. The concentration of As was slightly lower than those of Cu and Zn. The concentration of As in four tissues was around 40 μg/g. The concentration of Cd exceeded 5 μg/g in four tissues and was approximately 20 μg/g in the digestive glands. The concentration of Cr in the four tissues was below 1 μg/g and the concentration of Co was around 0.1 μg/g. The concentrations of Cr and Co were the highest in the gills in comparison with the other tissues. The concentration of As in the four types of tissue was basically the same, except that the concentration of As in the mantle was slightly higher than that in the other tissues.

The results of the Shapiro–Wilk test and the variance homogeneity test (F-test) indicated that the concentrations of the six elements in each tissue did not follow a normal distribution, nor did they meet the condition of variance homogeneity. Therefore, Kruskal–Wallis H test was performed. The distributions of Cr, Co, Cu, Zn, and Cd across the four tissues showed significant differences (*p* < 0.05). The distribution of As across the four tissues showed no significant difference.

### 3.2. Trace Elements Load Level in Each Tissue and Correlation of Trace Elements Distribution

**Figure 3:**
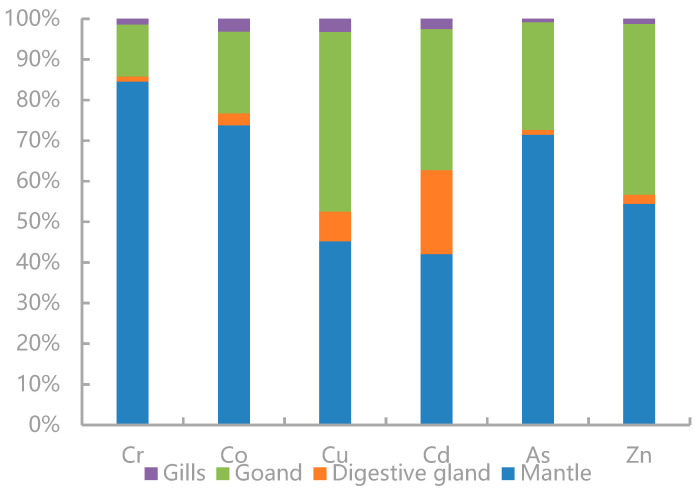
Total body burdens of Cr, Co, Cu, Cd, As, and Zn in the mantle, digestive gland, gonads, and gills of *Uroteuthis edulis*.

The proportions of the trace elements’ loads in each tissue of *U. edulis* were calculated (Figure 3). TEs were mainly accumulated in the mantle. The loads of Cr, Co, As, and Zn in the mantle accounted for more than 50% of the corresponding total loads and displayed the most significant enrichment, followed by those in the gonads. The proportions of the trace elements’ loads in the digestive glands were lower. The loads of Cu and Cd in the digestive glands were higher. Especially, the Cd load in the digestive glands was much higher than that in the other tissues and was significantly higher than the loads of the other elements in the digestive glands. The load proportions of all elements in the gills were the lowest.

A correlation coefficient between 0.3 and 0.5 indicates a weak correlation. A correlation coefficient between 0.5 and 0.8 indicates a moderate correlation; a correlation coefficient above 0.8 indicates a strong correlation. The absolute values of the correlation coefficients of Cr in the four tissues were all less than 0.3, indicating that the concentrations of Cr in the four tissues had no correlation. The correlation coefficient of Co in the digestive gland and gills was 0.459, which indicated a moderate correlation. The correlation coefficients of Cu among different tissues (mantle and gonads; mantle and gills; digestive gland and gills; gonads and gills) were, respectively, 0.381, 0.623, 0.531, and 0.388, which were all positive correlations. Cd only showed a medium correlation between mantle and gills, and the correlation coefficient was 0.566. The distribution of As in various tissues was quite uniform and remains stable (Table 2). The distribution of As in the four tissues showed moderate positive correlations.

**Figure 4:**
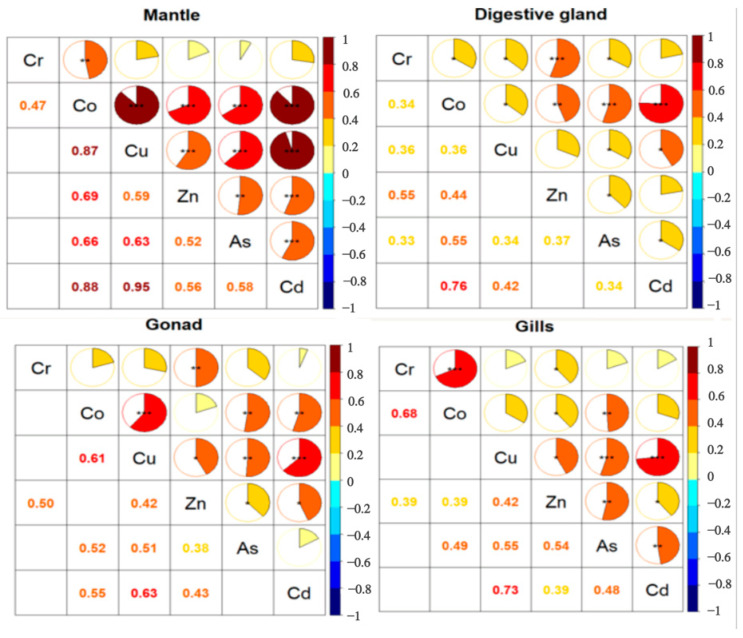
Correlation analysis of TEs concentration in various tissues of *Uroteuthis edulis*. Notes: *: *p* < 0.05; **: *p* < 0.01; ***: *p* < 0.001.

Through Spearman’s correlation analysis, we found correlations between the concentrations of different elements in the same tissue (Figure 4). In the mantle, with the exception where Cr showed no correlation with Cu, Zn, As, or Cd, the elements showed positive correlations with each other. In addition, Co showed a strong correlation with Cu and Cd; Cu also had a strong correlation with Cd. Their correlation coefficients were all above 0.8. In the digestive gland, strong positive correlations were found among the following TEs: Co and Zn; Co and As; Co and Cd; Cr and Zn. But the correlations among the other elements were not significant. The correlations of all the elements in the gonads and gills were not strong. In the gonads, Cu showed a significant positive correlation with Co and Cd. In the gills, significantly positive correlations were found among the following TEs (Cr and Co; Cu and As; Cu and Cd). The possibility that the correlations between the elements suggests that they have a common origin has not been confirmed.

### 3.3. Transfer Characteristics and Influencing Factors of Trace Elements in U. edulis

The concentrations of Cr in all four tissues except the mantle slightly decreased after maturity. The concentration of Co stayed almost constant in the mantle and the gonads and decreased in the digestive glands and the gills. The concentrations of Cu in the four tissues decreased after maturity. The decrease was the smallest in the mantle and gills and more significant in the digestive glands. Similarly, the concentration of Zn significantly decreased in the digestive glands, remained stable in the mantle and gonads, and decreased in the gills. The concentrations of As in all tissues decreased after maturity. The concentrations of As in both the digestive glands and the gonads greatly decreased. The concentration of Cd was the highest in the digestive glands in comparison with the other tissues and decreased after maturity.

The Shapiro–Wilk test and variance homogeneity test (F-test), carried out to determine the concentrations of the trace elements in various tissues of *U. edulis*, indicated that the concentrations of the six elements in the tissues did not follow a normal distribution, nor did they meet the condition of variance homogeneity. Additionally, for the two sample groups (immature and mature gonads), the Mann–Whitney U test was performed to test the differences (Table 3). In the mantle muscle, the concentrations of all the elements showed no significant differences after maturity (*p* > 0.05). However, in the digestive glands, all the elements except Zn showed no significant difference (*p* < 0.05). The correlations of the concentrations of all the elements in the gonads were rather complex. The concentrations of Cr, Cu, and As showed significant differences, but the concentrations of Co, Zn, or Cd showed no significant difference. In gills, the concentrations of Co and As showed significant differences, whereas the concentration of Cr, Cu, Zn, or Cd showed no significant difference.

In this paper, the transfer of trace elements in the gonads of *U. edulis* is mainly to verify the transfer efficiency of trace elements to offspring and their potential impact, which still requires further research in the future.

## 4. Discussion

### 4.1. Arsenic

Arsenic enrichment is unique. Arsenic is a nonessential element in human beings [29,30]. However, in aquatic species, Arsenic is a component of arsenobetaine, which has a similar structure to trimethylglycine and allows an organism to remain unaffected by changes in water salinity. As a result, Arsenic is a vital nutrient for the majority of aquatic species [30,31,32,33,34]. Arsenic is a component of arsenobetaine; therefore, it is essential in all tissues of *U. edulis*. As a result, the distribution of As in the tissues of *U. edulis* demonstrated that the As concentrations in four tissues was similar and showed no significant difference among the different tissues. In addition, the distribution of As was related to the size and growth state of *U. edulis*. In this experiment, the distribution of As in all tissue samples followed a normal distribution, as reported by other experts [30]. The correlation analysis of As concentration in various tissues revealed strong correlations of As concentrations in different tissues. The concentration of As in tissues showed no significant difference and its load proportion in four tissues was almost equal to the proportion of wet weights of four tissues. However, due to the large mass of torso, its proportion was much higher. The correlations between As and other elements were weak and the correlation between Co and Cu in mantle was strong. Arsenic showed a specific maternal transmission pattern [30]. According to the variations in As concentrations in all tissues after the maturity of the gonads of *U. edulis*, the concentrations of As in four tissues decreased. With the exception of the mantle, the other three tissues showed considerable changes in the concentration of As during gonadal maturation. As a result, it was difficult to determine whether it could be transferred with the parent material after maturity.

The concentration of As in *U. edulis* in this study was higher than that in previous studies and close to that which has been found for other cephalopods in the East China Sea [34], such as *Sepia longipes*. It was much higher than that in giant marine cephalopods like *S. pteropus* and benthic octopuses like *O. hubbsorum* due to the sampling locations of *U. edulis*.

### 4.2. Chromium

The concentrations of Cr in the muscle, digestive gland, gonads, and gills were 1.11 ± 1.18 μg/g, 0.89 ± 0.70 μg/g, 0.34 ± 0.21 μg/g, and 1.44 ± 1.42 μg/g, respectively. In the previous study (Koyama et al., 2000), the gills were identified as the primary concentration tissue of TEs, as confirmed by the variance in Cr concentrations found in this experiment. The concentration of Cr in *U. edulis* in this study was significantly lower than that which has been found in some oceanic species (Table 4), such as *Sthenoteuthis pteropus* (Lischka et al., 2018), *Loligo sanpaulensis* [28], and *Nototodarus gouldi* [29]. The concentration of Cr *U. edulis* was slightly higher than that in octopuses and some oceanic species due to the differences in the distribution areas of the species. For example, *S. pteropus* was widely found in the marginal zone of Cape Verde [30], where subtropical circulation and recirculation intersected. The marine conditions in this area made it easier for nutrients in water to bind to Cd. Similarly, *N. gouldi* accumulated around upwind flow areas in southeast Australia. Ocean currents transported Cr from adjacent seas to their habitat and a large quantity of Cr accumulated in their bodies. The concentration of Cr was the highest in gills, although the proportion of Cr load in gills was negligible in terms of trace elements load in the four tissues. The proportion of Cr load in mantle was the highest among the four tissues and exceeded 80%, whereas that in gills and digestive glands was less than 5%. The difference was related to tissue mass. The higher the mass was, the larger the proportion of TEs load was. In this investigation, the concentrations of Cr in gonads and digestive glands decreased dramatically after maturity, indicating that Cr could be passed to offspring from mother. Le also discovered that Cr in squid juveniles was primarily derived from the maternal source [31]. The results demonstrated the significant correlation between Cr and Zn in digestive glands, as well as the significant correlation between Cr and Co in gills. Many studies suggested that Co was largely absorbed and stored via gills in seawater [31,32].

### 4.3. Cobalt

The concentration of Co was the lowest among the six elements detected in this experiment. Among the four tissues, the gills contained the highest concentration of Co, at 0.15 ± 0.013 μg/g. The concentration of Co in the digestive glands was 0.12 ± 0.17 μg/g. The concentrations of Co in the mantle and gonads were relatively low, at only 0.05 ± 0.05 μg/g and 0.03 ± 0.01 μg/g, respectively. The gills were found to be the primary Co enrichment tissue due to their direct interaction with seawater, containing Copper. Bustamante discovered that the concentration of Co in the tissues directly in contact with Co-rich seawater significantly decreased after 6 days of contact with purified seawater [33], further demonstrating that squids interacted with Co through a mechanism involving seawater, and that the digestive gland was the primary organ of Co uptake. Due to potent retention capacity of this organ, the Co concentration in the digestive glands was found to be relatively high. Additionally, the digestive gland in adult squids was responsible for 91% to 95% of the total Co load in the body [33]. The findings of previous studies were significantly different from the findings in the present study because various species have differently sized digestive glands.

The concentration of Co in *U. edulis* was comparable to that in *Gonatus fabricii* and *Moroteuthopsis ingens* [30], but significantly lower than that in the pelagic cephalopods *S. pteropus* and Nototodarus sloanii [25,30]. The highest concentration of Co in the digestive gland of the benthic cephalopod *Octopus hubbsorum* reached 50 μg/g [34], which was significantly higher than the results in this experiment. In the sampling process of the above study, the *O. hubbsorum* was obtained in seriously polluted areas. As a benthic organism, *O. hubbsorum* was influenced not only by crustaceans and shellfish, with a strong TE-accumulation capacity, but also by Co pollutants in sediments; thus, the body contained an abnormally high concentration of Co. In other words, cephalopods are potentially at a risk of accumulating and absorbing Co through sediment.

Co showed a significant correlation with several elements (Cu, Cd, Cr, Zn, and As) in various tissues, particularly in the mantle. Co exhibited a high correlation with Cd in the digestive glands. The correlation might be ascribed to the relationship between Co and metallothionein [31]. Similar to Cu, Zn, and Cd, Co also induced metallothionein. As a result, in the mantle, a protein-rich tissue, Co was correlated strongly with some metals such as Cu, Zn, and Cd, which triggered metallothionein. This correlation could also be interpreted by the Co accumulation function of digestive glands. The digestive glands were found to contain a high concentration of metallothionein; Co can bind to metallothionein to perform its detoxifying function. Therefore, the concentration of Co in the digestive glands was found to be much higher than that in other tissues.

### 4.4. Copper

Cu, one of the most significant necessary elements in cephalopods, is abundant in all tissues. This is especially the case in the digestive glands, where the concentration of Cu was the highest. The concentrations of Cu in the tissues of different cephalopod species in different seas varied substantially. In this study, the Cu concentration in the digestive glands was 295.78 ± 677.72 μg/g, but the concentration of Cu in the digestive glands of squids found in the Gulf of Gabes, Tunisia, was only 3.538 ± 0.7 μg/g [35]. The concentration of Cu in the digestive glands of *O. hubbsorum* found in the Santa Rosalia region was the highest and reached 3296 μg/g [34]. As a result, regional pollutants and species’ habits have a significant impact on Cu concentration. Cu, as a component of hemocyanin, is a vital element for cephalopods. The digestive gland, as a digestive organ of cephalopods, also processes aged hemocytes. The hemocytes of cephalopods finally converge and are broken down in the digestive gland, thus resulting in a large quantity of Cu. Therefore, the determined concentration of Cu in the digestive gland was exceptionally high. The concentration of Cu in the gills was close to that in the digestive glands because they were both tissues (organs) through which a huge number of hemocytes passed. The concentration of Cu in gills was significantly positively correlated with that in the digestive glands.

In addition, only two Cu-containing proteins are involved in the transfer of nutrients from the carcass to the gonads: hemocyanin and metalloproteins. During the development phase of the gonads, the mantle delivers most of the nutrients to the gonads, but the concentration of Cu in the nutrients was found to be low. Therefore, the correlation between the mantle and the gonads was not significant.

Compared to the Cu accumulation exhibited by cephalopods in other seas, the concentration of Cu in the digestive glands of *U. edulis* in this study was higher; however, it was found to be lower than that in the benthic cephalopod, *O. hubbsorum*, in dense industrial areas, and *N. gouldi* in New Zealand waters. Compared to the findings of TEs accumulation in other cephalopods, the concentration of Cu in the digestive glands in this study was the highest due to the features of *U. edulis*. The mass proportion of the digestive gland in *U. edulis* was substantially less than that of other cephalopods. The correlation analysis of Cu and other elements in the four tissues revealed a strong correlation between Cu and Cd due to metallothionein features. Cu caused metallothionein to participate in Cd detoxification, thus resulting in a positive correlation.

### 4.5. Cadmium

In this study, Cd is the only nonessential element for aquatic organisms. Cephalopods feed on crustaceans and shellfish, both of which can enrich a large quantity of Cd, so the concentration of Cd in cephalopods was high. The concentration of Cd in the digestive glands of *U. edulis* was the highest (35.7 can c μg/g). The concentrations of Cd in the other tissues were similar. In related studies, the concentration of Cd in digestive glands was the highest regardless of the species or environmental conditions of the habitat. Cephalopods interacted with Cd mainly through ingestion. As a result, the concentration of Cd was directly related to the ingestion structure of cephalopods, changes in ingestion behaviors, and amounts of Cd in the habitat.

In comparison to previous studies, the Cd concentration in this study was lower than that of cephalopods in the East China Sea such as *Todarodes pacificus* and *Sepia madokai*, but higher than that of Gonatopsisborealis. The concentration of TEs in digestive glands was lower than in that in other seawater samples and the highest values were found in digestive glands of S. pteropus in the tropical eastern Atlantic. The concentrations of Cd in benthic cephalopods (*O. hubbsorum*) were higher than those in non-benthic cephalopods (*Sepia officinalis* and Gonatopsisborealis) because benthic cephalopods fed on more crustaceans and bivalves than non-benthic cephalopods. In addition, Cd adsorbed via different pathways had different retention durations in various tissues. Cd that was encountered via seawater had a biological half-life of around two months and TEs accumulated through foods had a half-life in tissues of several years.

Cd had a significant correlation with Cu and Co, particularly in the mantle. Cd had a moderate correlation with Zn. In previous studies, the correlations with the three elements were ascribed to metallothionein induction. However, Charles demonstrated that Zn was significantly correlated with Cd because Zn could more easily engage in the metallothionein reaction [31]. This viewpoint was contradictory with the correlation analysis results in this study. Cd load in four tissues was unique. The concentration of Cd in digestive glands was 5–15 times higher than in that in other tissues. Even though the mass fraction of a digestive gland was low, Cd load remained relatively high and reached approximately 20%. Cd load in the digestive gland of *Sthenoteuthis oualaniensis* was 72.18 ± 6.47% and Cd load in the digestive gland of *Nautilus macromphalus* in New Caledonia might reach 90%. The above results were significantly different from our study due to the differences in the mass of the digestive glands between various mollusk species.

### 4.6. Zinc

Both Cu and Zn are vital elements that are required during the growth process, so the concentration of Zn is higher than that of the other four elements. Different from Cu, Zn is most concentrated in the digestive glands and exists in the gonads (Figure 2). Zn is closely related to hemocyanin, which is abundant in the digestive glands, but the content of hemocyanin in the gonads is much lower, resulting in a significant difference between the two tissues. In comparison to previous studies on cephalopods in other oceans, the concentration of Zn in this study was in the medium–high level. The concentration of Zn in the digestive glands of *U. edulis* in the study was much lower than that in the benthic octopus due to the feeding patterns of benthic creatures. The concentration of Zinc in the gonads was much higher than that in other studies. Zn load in the gonads of *U. edulis* was only slightly lower than that in the mantle, indicating that the high concentration of Zn in the gonads was related to the proportion of nutrition transfer in different cephalopods.

### 4.7. Transfer Characteristics of TEs after Maturity

**Figure 5:**
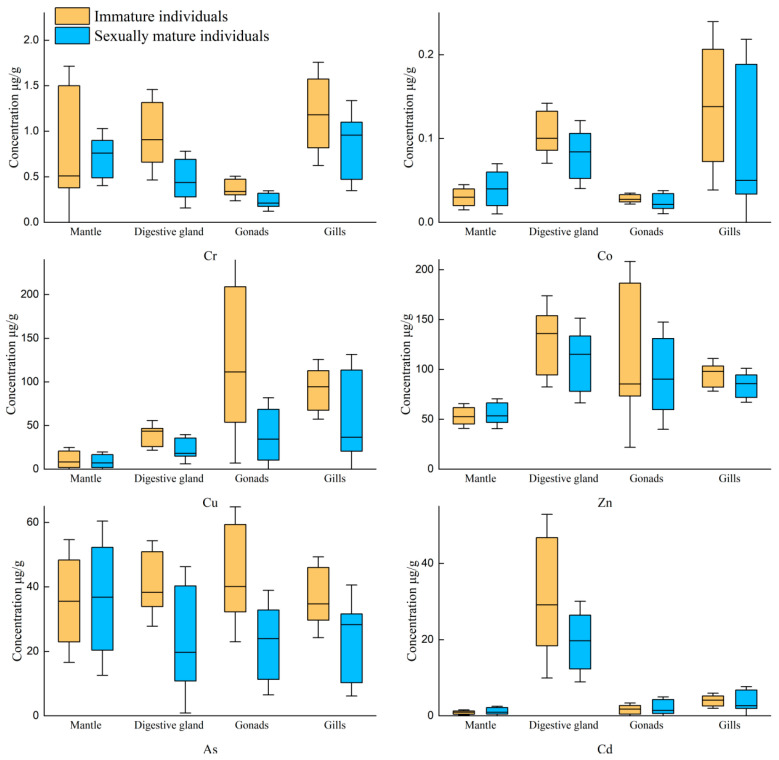
Comparison of the concentration changes of six TEs elements in various tissues of squid before and after gonadal maturation.

The mariculture conditions of *U. edulis* are not possible to study in the laboratory, so it was impossible to investigate the transfer characteristics of TEs in *U. edulis* with isotope tracing technology. As a result, the only feasible choice was to choose the samples of *U. edulis* in different life cycle stages and investigate the variations in TEs concentrations in various tissues. The Mann–Whitney U test was performed to investigate the factors that exhibited substantial differences in various tissues between mature and immature individuals (Figure 5).

Unexpectedly, the concentrations of various elements in the mantle showed no significant difference after gonadal maturity. As the tissue with the highest proportion of TEs load, the mantle should receive the most nutrients given by the muscles, thus resulting in a significant reduction in TEs. In *U. edulis*, more nutrients were externally obtained and the decrease in the concentrations of TEs in mantle was insignificant. In addition, the muscle mass was quite high. Even if more nutrients were transferred from muscle to gonads, the decreases in the concentrations of TEs were not significant. Furthermore, among various TEs, only As had the highest concentration in the mantle. The other elements had only slight concentrations in the mantle. As a result, the overall loss of TEs during transfer was reduced, and the decreased concentrations were too small to be detected by our instruments. Therefore, the decreases in the concentrations were insignificant.

In the digestive glands, the changes in the concentrations of TEs, with the exception of Zn, were found to be significantly increased due to the increase in the intake and the trophic level during the ingestion process of the nutrients. As the intake increased, the exposure to TEs also increased, and more nutrients were transferred to the gonads. TEs were more enriched in the digestive glands. Therefore, the concentrations of Cr, Co, Cu, As, and Cd were significantly increased. In the gonads, the concentrations of Cr, Cu, and As were significantly increased after gonadal maturity. The essential elements for *U. edulis* were closely related to growth and development, so these elements showed higher accumulation. Although Zn is an essential element, its concentration did not change significantly after gonadal maturity. This phenomenon requires further analysis and research. Some scholars have hypothesized that Cr and As are maternally transferred. The present study theoretically supports this view because TEs could be transferred from other tissues to the gonads. Isotope tracing techniques are recommended in subsequent studies.

Co and As concentrations in the gills showed significant differences between mature and immature individuals and significantly increased after gonadal maturity. It was verified that Co was enriched by the gills, so its concentration significantly increased during the ingestion process. The main accumulation pathways of As in cephalopods included seawater, so the concentration of As increased after gonadal maturity, similar to the concentration of Co.

Cephalopods cannot be cultured or continuously observed. Therefore, we only assessed the concentrations of TEs in the samples from different life cycle stages and then predicted TEs transfer features based on the comparison. In addition, some cultivable species can also be used to create a toxicological model to investigate the transfer properties of different tissues. In the future, we will explore the enrichment of TEs in *U. edulis* from prey after gonadal development and investigate the role of ingestion in the changes in TEs concentrations.

## 5. Conclusions

This study focuses on *U. edulis* in the East China Sea and aims to investigate TEs accumulation characteristics and influencing factors. The distribution, inter-element, and inter-tissue correlations of Cr, Co, Cu, Zn, As, and Cd in the mantle, the digestive glands, the gonads, and the gills were investigated. TEs accumulation characteristics, transfer characteristics, and influencing factors in the tissues of *U. edulis* in the East China Sea were determined. The key findings of this paper are summarized here.

The distribution of TEs in the tissues of *U. edulis* showed considerable heterogeneity. The digestive glands contained the highest concentrations of Cu, Zn, and Cd; thus, it was confirmed that the three metals accumulated mostly through consumption. The concentrations of Cr and Co were the highest in the gills, indicating that they mostly accumulated through the seawater. The concentration of As showed no significant difference among tissues. The TEs load was the highest in the mantle, followed by the gonads, whereas TEs loads in the digestive glands and gills were the lowest.

Some TEs found in the tissues of *U. edulis* differed significantly between mature and immature individuals. After sexual maturity, TEs in mantle decreased slightly. The concentrations of TEs except Zn in digestive glands increased dramatically. After maturity, gonads showed significant increases in the concentrations of Cr, Cu, and As. The concentrations of Co and As in gills increased significantly after maturity. Considerable specific components were transported from digestive glands and gills to gonads after maturity. In this study, we investigated the distribution of six trace elements in *U. edulis* samples and assessed their transfer before and after gonadal maturation, providing basic data for food health risk assessment, which is beneficial for social stability. In the future, more species should be subjected to long-term monitoring.

## Figures and Tables

**Table 1:** Concentration of heavy metals (μg/g) in scallop (GBW10024) obtained in the present study, showing the certified values and the recovery% (mean ± SD, n = 6).

Element	Certified	Determined	Recovery%
As	3.6 ± 0.6	3.35 ± 0.5	93.1
Co	0.047 ± 0.006	0.043 ± 0.12	91.5
Cu	1.34 ± 0.18	1.24 ± 0.09	92.5
Cd	1.06 ± 0.10	1.00 ± 0.03	94.3
Cr	0.28 ± 0.07	0.28 ± 0.1	99.9
Zn	75 ± 3	68 ± 4	90.7

**Table 2:** Trace element correlations among the tissues of *Uroteuthis edulis*.

Elements	Correlations
Cr	-
Co	+D and Gi **^b^
Cu	+M and Go *^c^ +M and Gi ***^b^ +D and Gi ***^b^ +Go and Gi *^c^
Zn	+D and Gi ***^b^
As	+M and D ***^b^ +M and Go **^b^ +M and Gi ***^b^ +D and Go **^b^ +D and Gi ***^b^ +Go and Gi **^b^
Cd	+M and Gi ***^b^

Notes: “+” denotes a positive correlation; *: *p* < 0.05; **: *p* < 0.01; ***: *p* < 0.001; M: mantle; D: digestive gland; Go: gonads; Gi: gills; ^b^: middle correlation; ^c^: weak correlation.

**Table 3:** Mann–Whitney U test of trace elements concentrations in mature and immature *Uroteuthis edulis*.

Groups	Tissues	Cr	Co	Cu	Zn	As	Cd
Mature	mantle	0.51 (0.38~1.50)	0.03 (0.02~0.04)	20.82 (8.25~27.16)	52.69 (45.31~61.78)	35.58 (22.95~48.34)	0.93 (0.40~1.29)
Immature		0.76 (0.49~0.90)	0.04 (0.02~0.06)	16.58 (7.13~33.87)	53.41 (46.86~66.46)	36.83 (20.39~52.29)	0.93 (0.47~2.17)
Z		−0.371	−0.591	−0.644	−0.124	−0.237	−0.241
p		0.725	0.569	0.534	0.915	0.827	0.825
Mature	digestive gland	0.91 (0.67~1.32)	0.1 (0.09~0.13)	209.08 (111.48~297.3)	136.03 (94.54~153.90)	38.31 (33.9~50.95)	29.13 (18.40~46.77)
Immature		0.44 (0.28~0.69)	0.08 (0.05~0.11)	68.67 (34.36~258.41)	115.15 (77.97~133.51)	19.67 (10.8~40.3)	19.72 (12.31~26.43)
Z		−3.199	−2.326	−2.102	−1.749	−2.904	−2.045
p		0.001	0.019	0.036	0.083	0.003	0.049
Mature	gonads	0.34 (0.30~0.47)	0.03 (0.02~0.03)	46.69 (43.59~88.75)	85.46 (73.35~186.43)	40.12 (32.28~59.37)	1.76 (0.46~2.73)
Immature		0.21 (0.18~0.32)	0.02 (0.02~0.03)	35.55 (18.03~71.71)	90.34 (59.69~131.06)	23.95 (11.32~32.85)	1.45 (0.64~4.25)
Z		−2.700	−1.102	−2.069	−1.115	−3.413	−0.084
p		0.006	0.283	0.047	0.285	0.000	0.948
Mature	gills	1.18 (0.82~1.57)	0.14 (0.07~0.21)	112.88 (94.30~123.70)	97.98 (82.32~103.36)	34.73(29.69~46.02)	4.1 (2.58~5.21)
Immature		0.96 (0.47~1.10)	0.05 (0.03~0.19)	113.64 (36.51~149.25)	85.69 (71.97~94.47)	28.26 (10.28~31.61)	2.66 (1.92~6.76)
Z		−0.976	−2.320	−0.973	−1.550	−2.451	−0.541
p		0.345	0.020	0.345	0.127	0.013	0.606

**Table 4:** Concentration of TEs (μg/g) of cephalopods.

Species	Tissues	Cr	Co	Cu	Zn	As	Cd	Location	Reference
*Uroteuthis edulis*	Mantle	1.11 ± 1.18	0.05 ± 0.05	37.01 ± 72.74	58.45 ± 19.05	36.99 ± 20.78	2.51 ± 5.54	East China Sea	This paper
	Digestive gland	0.89 ± 0.7	0.12 ± 0.17	295.78 ± 677.7	142.81 ± 154.21	43.37 ± 65.12	35.7 ± 59.63		
	Gonads	0.34 ± 0.21	0.03 ± 0.01	64.28 ± 56.77	120.8 ± 86.83	31.81 ± 17.57	2.98 ± 3.46		
	Gills	1.44 ± 1.42	0.15 ± 0.13	109.91 ± 55.04	89.07 ± 21.28	28.78 ± 12.75	4.44 ± 3.00		
*Todarodes pacificus*	Digestive gland	-	-	858	160	27	80.3		[29]
*Sepia longipes*	Digestive gland	-	-	48	320	113	34.3		
*Sepia madokai*	Digestive gland	0.60	-	1870	480	99	110.8		
*Ommastrephes pteropus*	Digestive gland	1.75 ± 2.13	19.9 ± 15.4	152.00 ± 206.0	187.00 ± 111.00	18.30 ± 11.70	748.00 ± 279.00	Eastern Tropical Atlantic	[25]
*Moroteuthopsis ingens*	Mantle (male)	0.13 ± 0.06	0.07 ± 0.04	11.79 ± 7.46	69.16 ± 15.05	12.48 ± 2.58	0.99 ± 0.75	Chatham Rise	[30]
	Digestive gland (male)	0.12 ± 0.03	0.17 ± 0.11	28.38 ± 34.92	42.65 ± 12.25	12.59 ± 2.03	116.00 ± 267.00		
	Mantle (female)	0.15 ± 0.13	0.06 ± 0.02	8.37 ± 4.05	66.88 ± 12.30	13.66 ± 3.40	0.57 ± 0.51		
	Digestive gland (female)	0.11 ± 0.03	0.15 ± 0.13	29.84 ± 63.76	44.55 ± 26.80	11.10 ± 1.71	52.90 ± 103		
*Nototodarus gouldi*	Mantle	0.26 ± 0.13	0.50 ± 0.65	25.4 ± 18.3	64.2 ± 8.89	29.9 ± 17.7	0.81 ± 0.51	New Zealand waters	[30]
	Digestive gland	5.00	1.99 ± 1.31	1185 ± 1008	351 ± 307	29.4 ± 14.3	194 ± 214		
*Nototodarus sloanii*	Mantle	0.32 ± 0.36	0.36 ± 0.56	23.3 ± 18	55.4 ± 11.8	11.9 ± 9.54	0.65 ± 0.46		
	Digestive gland	0.35 ± 0.26	0.89 ± 0.87	352 ± 581	77.7 ± 68.6	11.3 ± 5.08	89.3 ± 86.3		
*Gonatus fabricii*	Mantle (male)	0.28	0.03	18.80	54.00	26.30	-	Disko Island	[30]
	Digestive gland (male)	0.12	0.12	138.00	56.50	10.25	31.79		
	Mantle (female)	0.32	0.04	26.30	90.60	22.31	-		
	Digestive gland (female)	0.10	0.17	124.00	74.00	10.18	31.57		
	Mantle (young)	0.99	0.06	11.50	91.70	6.06	-		
	Digestive gland (young)	1.68	0.29	14.10	136.00	6.68	41.60		
*Uroteuthis sibogae*	Mantle	-	-	-	-	1.10 ± 0.43	0.187 ± 0.30	Manaar Bay	[32]
	Digestive gland	-	-	-	-	2.66 ± 1.56	7.33 ± 9.00		
	Gills	-	-	-	-	2.02 ± 0.80	0.754 ± 0.80		
*Sepia pharaonis*	Mantle	-	-	-	-	9.19 ± 10.6	0.055 ± 0.03		
	Digestive gland	-	-	-	-	8.02 ± 6.61	69.9 ± 30.8		
	Gills	-	-	-	-	7.17 ± 4.93	0.295 ± 0.19		
*Octopus hubbsorum*	Mantle	<0.12	<0.15	25	58	37	<0.13	Santa Rosalia	[34]
	Digestive gland	0.24	50	3296	877	43	76.00		
	Gills	0.28	2.20	136	87	33	0.70		
	Mantle	<0.12	<0.15	20	64	65	<0.13	La Paz	
	Digestive gland	0.49	6.00	2104	802	46	53.00		
	Gills	0.24	0.2	130	80	47	0.40		
*Sepia officinalis*	Mantle	-	-	0.115 ± 0.03	15.409 ± 3.9	-	0.153 ± 0.019	Gabes Bay, Gilbert Island	[35]
	Digestive gland	-	-	3.538 ± 0.7	2.7 ± 0.5	-	0.043 ± 0.02		
	Gonads	-	-	0.732 ± 0.2	10.724 ± 1.9	-	0.101 ± 0.04		
	Gills	-	-	3.041 ± 0.7	1.04 ± 0.2	-	0.032 ± 0.01		
	Mantle	-	-	0.45 ± 0.03	20.814 ± 7.6	-	0.185 ± 0.06	Gabes Bay, Gargour	
	Digestive gland	-	-	12.144 ± 2.4	4.397 ± 1.1	-	-		
	Gonads	-	-	0.82 ± 0.5	27.048 ± 7.3	-	0.272 ± 0.04		
	Gills	-	-	2.06 ± 0.63	1.646 ± 0.4	-	0.091 ± 0.02		

## Data Availability

The data presented in this study are available on request from the corresponding author due to data disclosure is at the discretion of the author of the communication.
